# The Effects of Zinc on Proprioceptive Sensory Function and Nerve Conduction

**DOI:** 10.3390/neurosci4040025

**Published:** 2023-11-10

**Authors:** Elizabeth R. Elliott, Kaitlyn E. Brock, Alaina C. Taul, Artin Asadipooya, Devin Bocook, Tessa Burnette, Isha V. Chauhan, Bilal Chhadh, Ryan Crane, Ashley Glover, Joshua Griffith, JayLa A. Hudson, Hassan Kashif, Samuel O. Nwadialo, Devan M. Neely, Adel Nukic, Deep R. Patel, Gretchen L. Ruschman, Johnathan C. Sales, Terra Yarbrough, Robin L. Cooper

**Affiliations:** Department of Biology, University of Kentucky, Lexington, KY 40506-0225, USA; erel222@uky.edu (E.R.E.); kaitlynbrock@uky.edu (K.E.B.); alaina.taul@uky.edu (A.C.T.); artin.asadipooya@uky.edu (A.A.); devin.bocook@uky.edu (D.B.); tessa.burnette@uky.edu (T.B.); isha.chauhan@uky.edu (I.V.C.); bilal.chhadh@uky.edu (B.C.); ryan.crane@uky.edu (R.C.); ashley.glover@uky.edu (A.G.); joshua.griffith@uky.edu (J.G.); jayla.hudson@uky.edu (J.A.H.); hassan.kashif@uky.edu (H.K.); samuel.nwadialo@uky.edu (S.O.N.); devan.neely@uky.edu (D.M.N.); anuukc233@uky.edu (A.N.); deep.patel1@uky.edu (D.R.P.); gretchenruschman@uky.edu (G.L.R.); johnathan.sales@uky.edu (J.C.S.); tmone.yarbrough@uky.edu (T.Y.)

**Keywords:** conduction, crustacean, proprioception, recruitment, sensory, zinc

## Abstract

Zinc (Zn^2+^) is an essential element that can promote proper organ function, cell growth, and immune response; it can also, however, be present in too great a quantity. Zinc toxicity caused by overexposure may result in both minor and major physiological effects, with chronic exposure at low levels and acute exposure at high levels being harmful or even toxic. This investigation examines the effects of acute exposure to relatively high concentrations of Zn^2+^ on sensory nerve function and nerve conduction. A proprioceptive nerve in marine crab (*Callinectes sapidus*) limbs was used as a model to assess the effects of Zn^2+^ on stretch-activated channels (SACs) and evoked nerve conduction. Exposure to Zn^2+^ slowed nerve condition rapidly; however, several minutes were required before the SACs in sensory endings were affected. A depression in conduction speed and an increase followed by a decrease in amplitude were observed for the evoked compound action potential, while the frequency of nerve activity upon joint movement and stretching of the chordotonal organ significantly decreased. These altered responses could be partially reversed via extensive flushing with fresh saline to remove the zinc. This indicates that subtle, long-term exposure to Zn^2+^ may alter an organism’s SAC function for channels related to proprioception and nerve conduction.

## 1. Introduction

The element zinc is essential for animals, serving as a cofactor for many enzymes and supporting the structural integrity of hundreds of other proteins (see reviews [1,2]). Current estimates suggest that zinc comprises 1.5 g of the average female’s body content, and 2.5 g of that of the average male; this represents, for a 70 kg male, about 0.004% by weight [3]. Past investigations report that humans generally occupy a state of zinc deficiency [1], and that dietary changes or oral supplementation can be used to rectify that lack. Red meat, pulses/legumes, and, albeit to a lesser extent, cereals represent major sources of dietary zinc [1], and various zinc additives are available over the counter. Unfortunately, the accessibility of zinc-rich food and vitamin- or pill-based supplements varies with culture and economy, as the affluent tend to have greater access to both than the relatively less wealthy [1], so insufficient zinc consumption continues to be an ongoing problem.

While this zinc insufficiency is concerning, however, the overuse of oral zinc supplementation is also an issue, and deaths have been reported due to consuming it in excess [4,5,6]. Additionally, zinc exposure can also stem from industrial or occupational hazards, such as the airborne zinc particulates prominent in mining operations [7,8] or the accidental contamination of food or water from storage in galvanized containers or pipes [4]. These paths of exposure could affect populations nearby, whether through chronic or acute effects, and have health repercussions that are, as of now, not fully understood.

The mechanisms behind zinc toxicity are complex. Over time, excess zinc is known to compete for transport mechanisms with copper, another essential metal, and produce an overall deficiency [1]. Additionally, the direct effects of Zn^2+^ have been observed to affect ion channels [1,9], enabling its use in the treatment of diseases involving copper overload (i.e., Wilson’s disease), as well as excess diarrhea, pneumonia, sickle cell anemia, malaria, and even the excess loss of zinc that comes from diabetes [1,5,6,10,11]. The effects of excess free zinc ions are known to correlate with neurological issues, but zinc has also been discovered to play an important role in maintaining normal function in neurons, especially within the synaptic vesicles of nerve terminals (see review [9]). A correlation has been observed between excessive neural activity, such as that occurring during experimentally induced seizures in rodents, and higher free zinc levels in the extracellular fluid, though the exact origin of the zinc itself is debated [9,12,13,14].

The effects of excessive zinc on the nervous system have largely been observed affecting the central nervous system, while zinc deficiency is known to affect vision by the alteration of retinal dehydrogenase synthesis [15,16,17]. Zinc sulfate is known to act on the olfactory epithelium and alter the olfactory capability in both mammals and zebrafish [18,19,20], while it has been suggested that zinc dust might activate TRPA1 receptors in C-fibers of the rodent pulmonary system’s sensory nerves [21]. It would be of interest to investigate how zinc might affect the various subtypes of stretch-activated channels (SACs) found in sensory neurons, as SACs are associated with many functions: blood pressure, osmoregulation, mechanical deformation, proprioception, and pain.

Since zinc is essential for the normal development of the nervous system, as well as proper cellular function, and since excessive zinc can cause mitochondrial dysfunction, it would not be surprising for every cell within an animal to be affected by either a deficiency or an overload of zinc over time. However, it is also of interest to understand the effects of an acute (i.e., seconds’ or minutes’ worth) overexposure, and, in particular, how zinc affects the primary sensory neurons of various modalities and axonal electrical activity in general. Understanding these topics could lead to more information about zinc’s subtler effects, such as how low concentrations might affect cellular function over time.

Acute responses can also provide insight into the mechanisms behind prolonged exposure. In this investigation, the proprioceptive neurons in the blue crab walking legs were used as a sensory model to assess the function of acute exposure to ZnCl_2_ in sensory receptivity and electrical conduction along nerves. Insect and crustacean limb proprioception is largely carried out by chordotonal organs, which monitor the movement and static positions of the joints. The sensory endings found within these chordotonal strands contain SACs such that the movement of the strand results in mechanical–electrical coupling. Generally, information is relayed to an animal’s central nervous system using both dynamic and static displacement-sensitive neurons [22,23,24,25,26,27]. Large crabs, like the common blue crab in question (*Callinectes sapidus*) or the Dungeness crab (*Cancer magister*), feature a nerve of approximately ten to fifteen centimeters, located from the most distant chordotonal organ, the PD organ (the propodite-dactylopodite organ), to the base of the thorax; this allows for the easy examination of electrical conduction along the nerve, as well as an investigation into how exposing the nerve to different baths (and, within the baths, different agents) could affect this conduction [28,29].

It is not yet known what types of SACs are located within the sensory endings of these chordotonal organs, as these have yet to be fully described via either pharmacological profile or mechano-electrical transduction properties. However, the subtype present in the crab chordotonal organs seems to maintain function without a need for Ca^2+^ in the bathing environment [28]. These channels are not affected by compounds that traditionally affect SACs, to the point that traditional antagonists and agonists—such as amiloride, ruthenium red, and/or streptomycin—do not function in their usual manners [30,31]. These SACs are also not altered by the selective compounds that affect SACs of the PIEZO 1 subtype (i.e., YODA 1, JEDI 2, OB 1, and DOOKU) [31]. Recent research has used crab proprioceptive organs as a marine neuronal model to examine the effects of heavy metal exposure, as well as to investigate various concepts of neurophysiology and to pharmacologically profile SACs [32,33,34,35,36]. The purpose of this experiment was to investigate how zinc (Zn^2+^) affects proprioceptive neural activity and nervous signal transmission.

## 2. Materials and Methods

The general procedures followed are similar to those previously described in detail [28,29,33] and in a video format [37].

### 2.1. Animals

Blue crabs (*Callinectes sapidus*) were obtained from a local supermarket in Lexington, KY, where they had been delivered from a distribution center in Atlanta, GA. They were bought and maintained in a seawater aquarium for several days prior to use so that their health could be assessed. Adult crabs of 10–15 cm in carapace width (from point to point) were used, and all were active when the leg was autotomized for experimentation.

### 2.2. Dissection and Physiology

**Figure 1:**
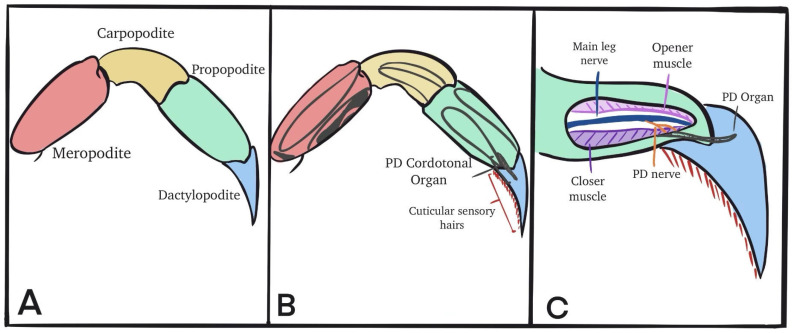
The walking leg of the crab, with identification of the PD organ’s location. (**A**) The segments of the overall leg are shown. (**B**) The last two segments are shown to house the PD organ. (**C**) The PD nerve is identified as the branch from the main leg nerve to the PD chordotonal strand.

Each crab had their first or second walking leg lightly pinched with pliers at its base to induce autotomization. The segments of the walking leg are identified (Figure 1A). The apodems to which the muscle within each segment attaches are shown (Figure 1B). The propodite–dactylopodite (PD) chordotonal organ spans the last segment of the leg (Figure 1C), and was exposed by cutting a window into the cuticle on both sides of the leg (in the propodite segment).

**Figure 2:**
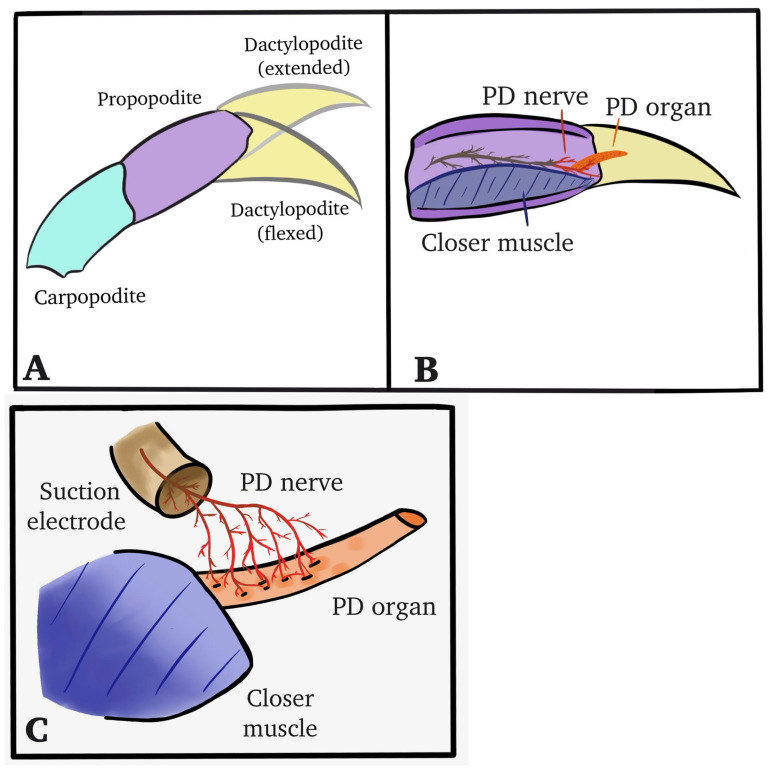
The distal PD joint and displacement range while PD nerve activity was monitored. (**A**) The PD joint was held in the flexed position before being displaced to an extended position and held there to stimulate the dynamic and static position-sensitive neurons. (**B**) The PD nerve is depicted as isolated from the main leg nerve. (**C**) The isolated PD nerve is pulled into a suction electrode to record neural activity during displacement of the joint.

Once a window into the cuticle had been established, the PD nerve could be observed independently from the main leg nerve (Figure 1C). The chordotonal organ spans the PD joint. After the windows were made and the cuticle removed, the leg was pinned in a Sylgard-lined dish and bathed in saline (Figure 2). The standard crab saline used during recordings of the sensory nerves consisted of (in mM): 470 NaCl, 7.9 KCl, 15.0 CaCl_2_·2H_2_O, 6.98 MgCl_2_·6H_2_O, 11.0 dextrose, 5 HEPES (4-(2-hydroxyethyl)-1-piperazineethanesulfonic acid) acid, and 5 HEPES base, with the solution adjusted to pH 7.5. To examine the effect of ZnCl_2_, the compound was dissolved directly in the crab saline with no substitution of the salts.

The datylopodite was moved from a flexed position to an extended one across a single second, held for at least ten more, and then moved back to the starting position (Figure 2A). An insect dissection pin was used to mark the maximum displacement range to ensure consistency across the trials, and the beginning of each displacement was marked on the recorded computer file with a comment. The PD nerve had been exposed and pulled into a suction electrode (Figure 2B) to allow for the recording of the activity while the PD joint was manipulated (Figure 2C).

**Figure 3:**
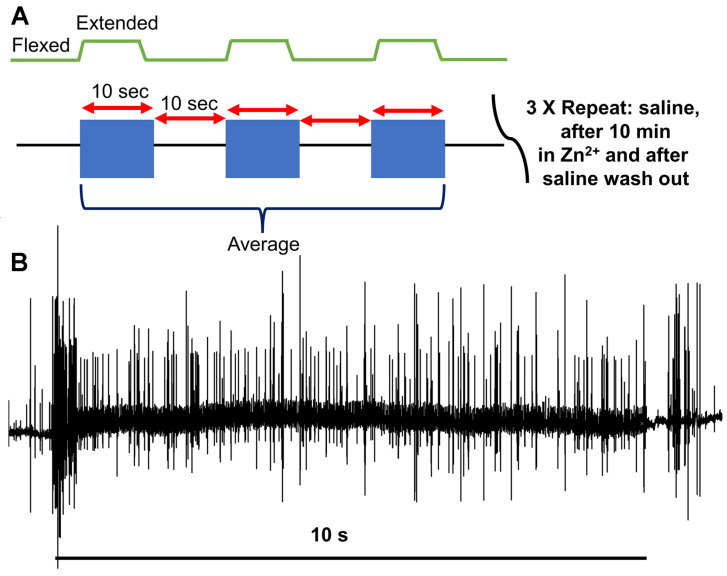
The experimental paradigm for joint displacement and analysis of the spikes. (**A**) The joint was displaced from a flexed position to an extended position during a single second, held in the extended position for at least ten, and then moved back to a flexed position. This was repeated thrice for each bathing solution. (**B**) The number of spikes observed from the beginning of the movement (the first second) through the next nine (when it was held static in an extended position) was used as an index of neural activity for the PD organ. The average number of measured spikes across each of the three trials was used as a metric of neural activity in that condition.

The index used to monitor changes in neural activity was established by counting the number of extracellular recorded action potentials (i.e., spikes) recorded across the first ten seconds upon joint displacement. In each condition, the displacement occurred thrice, with ten seconds between each. The numbers of spikes from the three trials were averaged to allow for graphing and comparison across the conditions (Figure 3).

**Figure 4:**
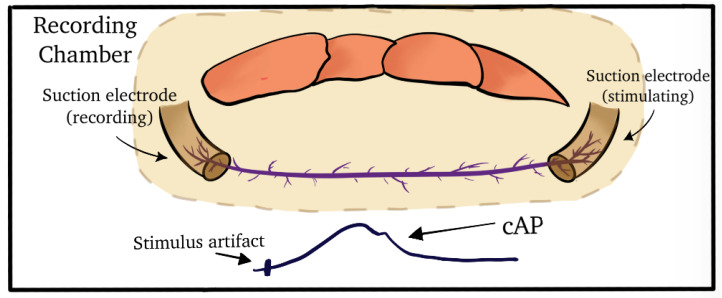
Recording compound action potentials (CAPs) of the PD nerve. The PD nerve was dissected along the length of the main leg nerve to the cut end of the meropodite. The nerve was placed in a saline bath for recording. Each end of the nerve was pulled into a suction electrode for induction and recording of the CAPs. The bathing medium was exchanged from saline to the compounds of interest and back to fresh saline over the course of the experiment.

The PD nerve was dissected from the proximal end of the meropodite to the PD organ. The nerve was transected next to the PD organ to remove the sensory endings, ensuring that only stimulus-evoked responses would be induced, allowing good recording of the compound action potentials (CAPs) along the nerve (Figure 4). In this arrangement, stimulating one end of the PD nerve initiates CAPs, which are then recorded at the other end. The PD nerve was exposed to compounds thoroughly while changing out the bathing media.

### 2.3. Statistical Analysis

All data are expressed as a mean (±SEM, standard error of the mean). The paired *t*-test was used to compare differences in response before and after exchanging solutions. The Shapiro–Wilk test was used for normality. When appropriate, the Wilcoxon rank sum non-parametric test was used. Analysis was performed using Sigma Stat software. A *p*-value of <0.05 was considered statistically significant for determining changes upon exposure to the compounds.

## 3. Results

**Figure 5:**
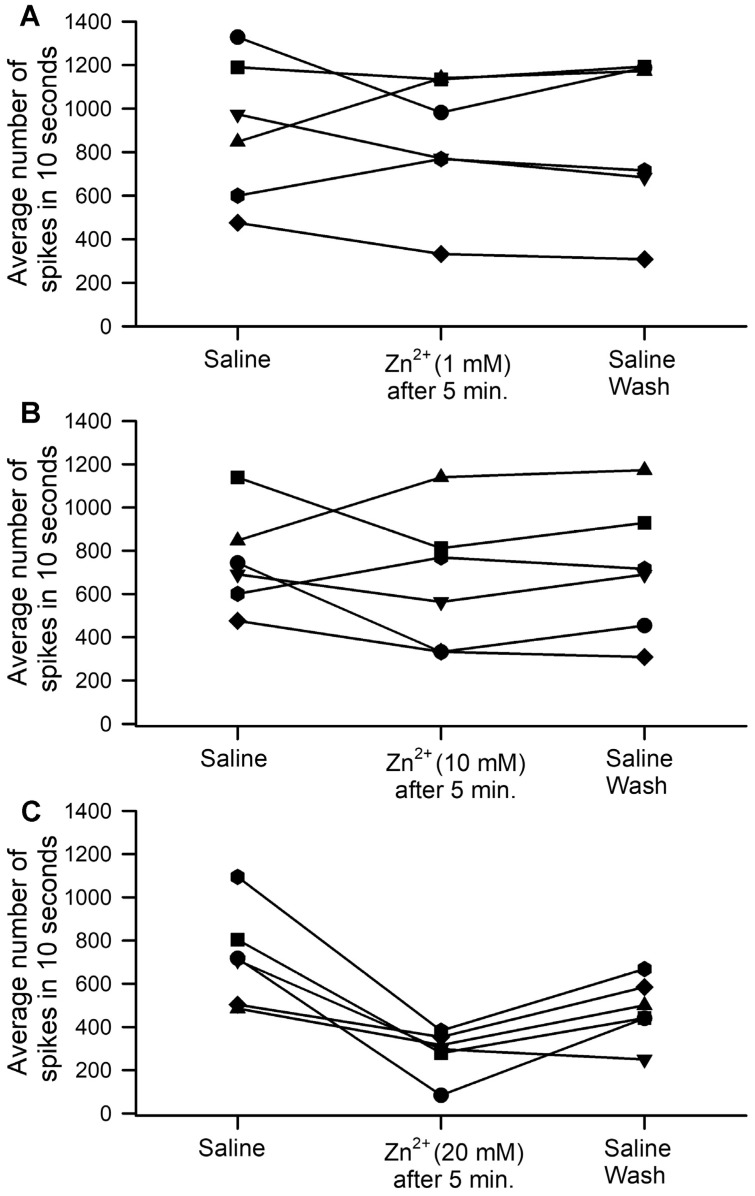
The effect of ZnCl_2_ on the PD activity with joint displacement. The responses in the nerve activity varied among preparations, with some increasing and others decreasing at both (**A**) 1 mM and (**B**) 10 mM concentrations (no significant differences were observed). (**C**) At 20 mM, there was a significant decrease in overall activity, which partially recovered with washout. Each line/shape on the above graph represents the results observed from an individual preparation.

The activity of the PD nerve before, during, and after (i.e., under washout conditions) five minutes of ZnCl_2_ incubation produced varied effects, both at 1 mM and at 10 mM (Figure 5A,B); however, exposure to 20 mM ZnCl_2_ produced a consistent decrease in activity and only some recovery upon washout (Figure 5C). No significant effects were observed upon five-minute acute exposure for either 1 mM or 10 mM ZnCl_2_ (*p* > 0.05; paired *t*-test, N = 6), but effects were observed at 20 mM (*p* < 0.05; paired *t*-test, N = 6 from initial saline to ZnCl_2_ and from initial saline to washout saline, as well as ZnCl_2_ to washout saline). This data indicates that the washout did not fully recover normal function in this paradigm for the 20 mM ZnCl_2_ exposure.

**Figure 6:**
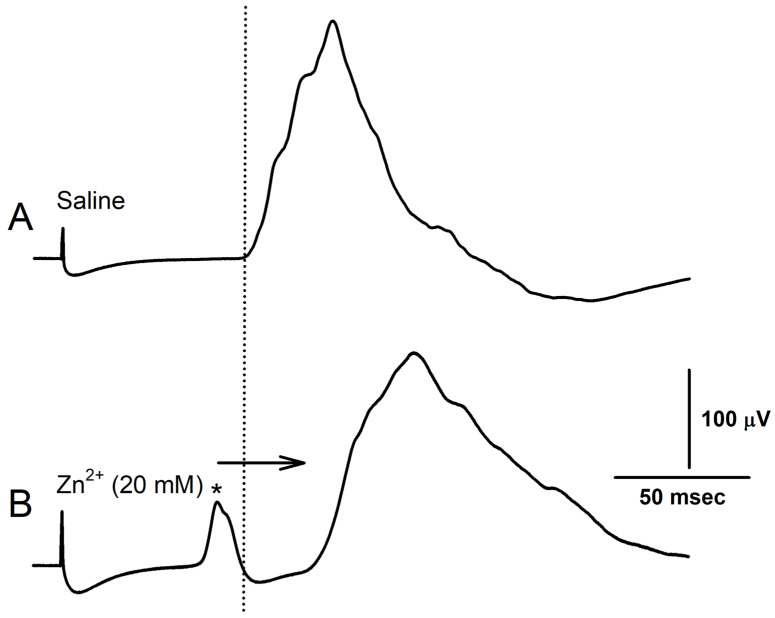
The effects of ZnCl_2_ exposure on the production of spontaneous nerve firing (*). The dotted vertical line is shown as a reference to help illustrate the shift in the conduction time when exposed to Zn^2+^. Note the arrow indicates the shift to the right of the main CAP before (**A**) and during the exposure to Zn^2+^ (**B**).The * indicates the recruitment of additional axons that were recruited by the zinc exposure.

**Figure 7:**
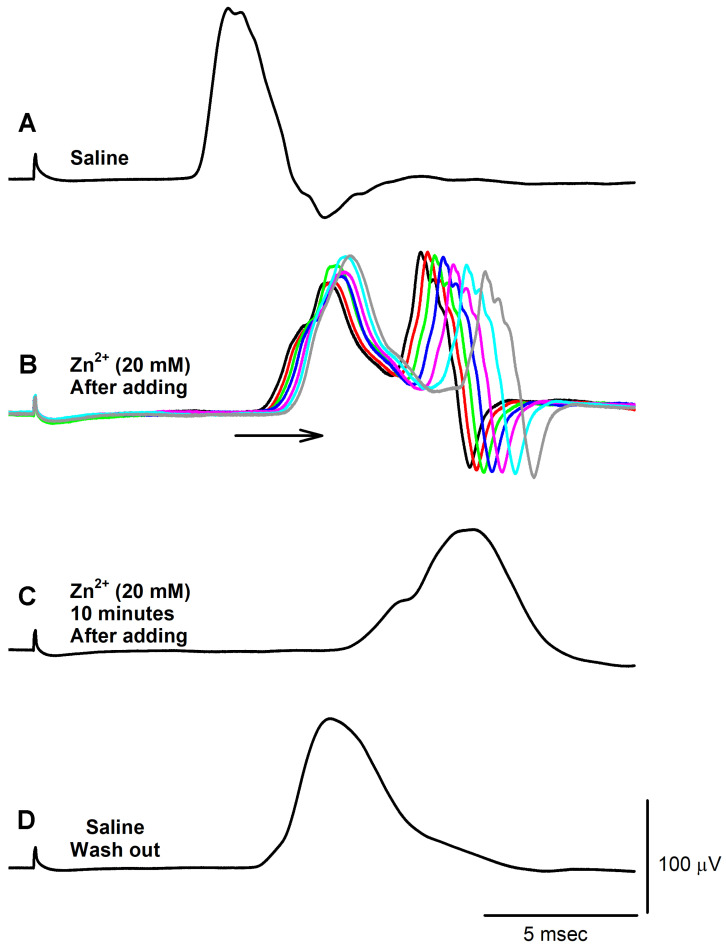
The acute effects of ZnCl_2_ exposure on CAPs, increasing the amplitude and slowing the conduction velocity. CAPs are shown as follows: (**A**) During saline-only exposure. (**B**) Immediately upon changing the bath to a 20 mM ZnCl_2_ solution, illustrating the amplitude increase and conduction velocity decrease. The arrow shows the direction of the shift to the right over time. Each superimposed trace was 20 s apart to visualize the rapid effect of ZnCl_2_ on the conduction speed. The colors were added to help visualize the separate traces. (**C**) After 20 min of ZnCl_2_ incubation, with CAPs of reduced amplitude, increased width, and still-slowed conduction velocity. (**D**) Upon removal of the ZnCl_2_, three washouts with fresh saline and the flushing of the nerve, with CAP shape and conduction velocity trending to a return to normal conditions.

The electrical excitation of the isolated nerve gave consistent changes, with conduction velocity slowing upon exposure to ZnCl_2_ (20 mM) immediately after switching the bathing medium. Some preparations were observed producing additional spontaneous nerve activity immediately after the addition of saline containing ZnCl_2_, as shown in Figure 6. CAP amplitude was also observed to rapidly increase before being followed by a smaller-amplitude CAP in the subsequent few minutes. The amplitude increases and conduction velocity delay can be observed by laying the CAPs overtop one another (Figure 7). However, after 10 min of ZnCl_2_ exposure, the CAPs were observed to undergo a decrease in amplitude and an increase in width as the overall shape was altered (Figure 7C). Removing the zinc and flushing the bath thrice with fresh saline to ensure thorough decontamination resulted in the conduction velocity speeding up and the width of the CAPs trending back towards baseline conditions (Figure 7D).

The trends observed relating ZnCl_2_ (20 mM) exposure to changes in CAPs for all six preparations were consistent (Table 1). Since nerve size varied in length across different types of crab and different leg spans, trend within each preparation is the best measure without overemphasizing the absolute changes. The amplitude of the CAPs increased (*p* < 0.05 paired *t*-test; N = 6) and then decreased (*p* < 0.05 paired *t*-test; N = 6) when exposed to Zn^2+^. The percent changes from the initial amplitude values are shown in Table 1. Over the 30 min of exposure, the amplitudes of the CAPs were the smallest with the longest delay in conduction time to the peak amplitude. In all preparations, there was an increase in condition time to the peak amplitude when exposed to Zn^2+^ over 30 min. The change in the time from the initial peak compared to the delay in the time to the peak is shown in Table 1.

**Figure 8:**
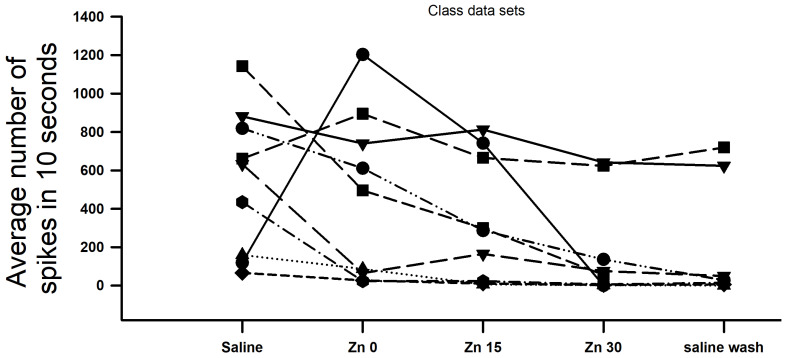
Effects of acute Zn^2+^ exposure on PD nerve activity across 30 min, as observed during joint displacement/extension by nine different groups of researchers. Furthermore, fourteen students used seven recording set-ups (two individuals per set-up) to confirm that CAPs from isolated nerves in the crab leg experienced both reduced conduction velocity and decreased amplitudes across 30 min of Zn^2+^ (20 mM) exposure, as shown above (N = 7, *p* < 0.05 paired *t*-test). Each line/shape on the above graph represents the results observed from an individual preparation.

To ensure the validity of the data, test the reproducibility of the observations, and avoid potential bias by any given investigator, students were recruited to externally verify the results. Nine groups of two students each carried out the same procedures of saline incubation, Zn^2+^ (20 mM) exposure, and general joint movements. These experiments were conducted at the same time, such that all data were comparable across the groups (e.g., using the same solutions, etc). As shown (Figure 8), the overall trends were consistent with the previously observed results: activity decreased over time when the bathing medium contained Zn^2+^ solution (N = 9, *p* < 0.05 paired *t*-test). These data might be less consistent than those gathered in a research lab, as the course participants may not have manipulated the joints as consistently as in the first set of experiments, wherein a single individual moved the joint for all data sets presented; because of this, the data sets are provided separately. Each preparation saw the overall activity decrease after 30 min of Zn^2+^ exposure. Seven preparations saw diminished success at returning to baseline under washout conditions; these were also the most affected by the exposure to zinc.

## 4. Discussion

This experiment demonstrated that acute exposure to ZnCl_2_ can affect primary sensory neurons—potentially by blocking stretch-activated channels (SACs) in sensory endings—and result in transient neuron recruitment to become active upon electrical stimulus. Neuron excitation was also observed to be followed by reduced excitation, as shown by the reduced CAP amplitudes, and ZnCl_2_ was also observed to rapidly result in reduced neuronal conduction velocity. While neither 5 nor 10 mM ZnCl_2_ solutions resulted in consistent effects on SACs during five minutes of exposure, these concentrations were shown to affect neuron recruitment: increasing, then decreasing, the CAP amplitude, and slowing conduction velocity. On the other hand, 20 mM ZnCl_2_ solutions rapidly depressed the overall activity, as shown by the neuron conduction velocity observed in conjunction with the joint displacement.

The mechanisms behind these effects on the PD organ are likely complex. Zn^2+^ may block SACs within the sensory endings, which would account for the activity decrease during the displacement and extension of the joint. Recordings from the PD nerve saw a very pronounced decrease in overall activity during joint displacement at 20 mM; however, stimulation of the isolated nerve to measure the CAPs led to well-pronounced amplitudes, indicating that the nerve is still functional and able to transmit any spikes that occur. Thus, it appears as though Zn^2+^ exposure may act through the retardation of the mechanical stimulus transduction to electrical events. Since the SACs appear to conduct Na^+^ rather than Ca^+^, Zn^2+^ could be blocking these Na^+^ SAC ionotropic channels more than the axonal voltage-gated Na^+^ channels, thus activating the action potentials and conducting electrical events, as observed in the CAP recordings.

The SACs in insect joints and crustacean chordotonal organs have yet to be classified, either pharmacologically or molecularly. This is quite surprising, as both insect and crustacean chordotonal organs are well-documented. Indeed, physiological recordings of a variety of these invertebrates indicate a wide variety of physiological subtypes. At present, SACs in the crab PD organ are believed to be of the DEG/ENaCs subtype, making them permeable to Na^+^ [38]. Previous investigations into bathing solutions absent added Ca^2+^ still allowed the PD organ to respond to joint movements [28]. Additionally, as previously mentioned, the PD organ was used to screen newly described pharmacological compounds (YODA 1, JEDI 2, OB 1 and DOOKU), SACs of the PIEZO 1 subtype; these were found to have no effect on the activity of the crab PD organ [31]. Other SAC subtypes may also be responsible for the sensory transduction in the crab PD organ, though further research is needed to identify them. The findings here, which might indicate that Zn^2+^ blocks these SACs, can help with pharmacologically profiling the subtype.

Given that ZnCl_2_ was found to depress neural activity during the movement of the PD joint, it is possible that the compound gains access to sensory endings within the scolopale of the chordotonal strand. Electron microscopy of these sensory endings in the PD organs of a different crab species illustrates the anatomical arrangement of the sensory ending within the scolopale structure [39].

The rapid deceleration in conduction velocity upon exposure to ZnCl_2_ suggests that one mechanism of the compound’s action is to affect voltage-gated channels; for example, by slowing down the recovery from depolarization, it would affect voltage-gated K^+^ channels, (potentially) block voltage-gated Ca^2+^ in the axon, and activate calcium-activated potassium channels (K_(Ca)_), resulting in a delay of membrane repolarization after activation. This would indicate that the neurons should remain depolarized longer after activation, even to the point that, rather than relying on evoked stimulation, spontaneous firing would be observed (should the membrane potential not return to baseline before further depolarization occurs). Please note that it has not yet been established whether the conduction delay is activity-dependent.

Another possible mechanism behind Zn^2+^ action may be the direct inactivation of Na^+^ voltage-gated channels, prolonging the inactive state by not allowing the conformational change necessary to remove that inactivation. While it seems unlikely that zinc might be acting directly on the membrane to influence capacitance, it is possible that the additional charge added by the Zn^2+^ could alter the discharge of the membrane in the traveling circuit along the axon.

To address these possible mechanisms, intracellular recording should be carried out in the axons to examine the shapes of the action potentials and the selective function of the ion channels. However, the axons are very small in diameter, and the sheath around the PD nerve would need to be peeled away, which has yet to be attempted. Thus, it might be better to address such electrophysiological matters by using *Callinectes sapidus* axons within the ventral nerve cord, or perhaps a large squid neuron, in order to obtain intracellular recordings that might simulate the axons of the PD nerve.

The extra activity observed in the nerve recordings while examining the effects of ZnCl_2_ on the CAPs may be due to Zn^2+^ blocking Ca^2+^ channels along the membrane; a similar phenomenon has been observed when reducing the Ca^2+^ content of a bathing medium, and it would make sense that, if Zn^2+^ blocks the Ca^2+^, the neuron would behave similarly to its action under low-Ca^2+^ conditions. It is well-known that low extracellular Ca^2+^ results in neuron hyperexcitability [40,41], but the mechanism is still not well understood [42,43,44,45,46,47]. While adding 20 mM ZnCl_2_ does increase osmolarity, changes of this degree are known to not alter PD activity or nerve conduction in the preparations. The osmolarity of crab saline is approximately 1041 Osm, and adding 1 M sucrose or 1 M mannitol to the normal crab saline did not have any effect on neural activity within the time frame used for these studies [33]. A similar phenomenon was recently reported for sensory neurons in crayfish, where an increase in nerve excitability occurred with exposure to ZnCl_2_ (1 mM) [48]. In addition, ZnCl_2_ at 0.1 mM reduced evoked synaptic transmission at the neuromuscular junctions of larval *Drosophila* and crawfish by blocking presynaptic, voltage-gated Ca^2+^ channels in the motor neurons [48]. In addition, ZnCl_2_ (0.5 mM) decreased the heartbeat in larval *Drosophila*, likely by blocking the plasma membrane voltage-gated Ca^2+^ channels [48].

Zn^2+^ is known to block certain subtypes of K2P channel, specifically those known as TREK-1 and TASK-3 [49]. This results in nerve depolarization and, depending on the extent of that depolarization, possibly the inactivation of voltage-gated Na^+^ channels, producing an overall decrease in neural activity. However, it is not currently known whether these Zn^2+^-sensitive K2p channels exist in the crab sensory neuron.

These results hold great significance in healthcare, industrial, and environmental contexts, as zinc is prevalent in those fields despite there being limited research on its mechanisms of action. In particular, little work has been done to investigate the connection between zinc and its effects on proprioceptive function; as such, research focused on this topic—like that described herein—is crucial. Understanding the effects of acute exposure could alter the current understanding of how chronic exposure affects an organism, as well as regulating workplace safety protocols and zinc supplement usage. Additionally, zinc seemingly affects sensory function, especially proprioception-related SACs in the muscle spindle. Regular exposure might thus compromise proprioception, leading to increased fall risk, among other effects.

To ensure the reproducibility of these results, an ACURE (authentic course-based undergraduate research experience) approach was utilized. Here, 18 students from a college neurophysiology course repeated the same experiments to simultaneously increase sample size and receive experience in an authentic research setting [29,48,50]. This approach builds on the CURE (course-based undergraduate research experience) concept by letting students develop experience in group research through a course setting. The students worked in groups to replicate the afore-described experiments, and their results followed the same trends. Despite the greater potential for error in the classroom setting—a lack of vibration-free tables, as well as an inability to ensure consistent speed at moving the joint—and differences in the absolute values observed, the analysis has indicated the same results.

## Figures and Tables

**Table 1:** The effects of zinc on compound action potentials (CAPs).

Prep	Amplitude of CAP	Neuronal Recruitment	Conduction Time (ms)	Recovery in Saline Rinse
1	↑ 44% then ↓ 88%	+	↓ 17.97 ms	+
2	↑ 11% then ↓ 30%	+	↓ 7.55 ms	+
3	↑ 3% then ↓ 39%	+	↓ 8.04 ms	+
4	↑ 134% then ↓ 14%	+	↓ 14.02 ms	+
5	↑ 20 then ↓ 29%	+	↓ 3.8 ms	+
6	↑ 303% then ↓ 50%	+	↓ 17.97 ms	+

The six preparations (i.e., preps) are listed, and the initial effects of ten minutes of zinc exposure on amplitude—an increase, represented by the upwards arrow (**↑**), and then a decrease shown by the downwards arrow (**↓**)—are indicated. The percent change in the amplitude of the CAPs was determined by % change = [(initial- experimental)/initial)] × 100. The random firing of the nerve producing the CAPs out of sync with the stimulus is indicated with a plus sign (+) to show recruitment of neurons. The reduction in conduction time across all preps is indicated by a downwards arrow (**↓**). The decrease in conduction time is relative to the peak of the initial to the slowest time measured prior to the condition velocity increasing when Zn^2+^ was removed. The trend of returning to initial baseline conditions is indicated by a plus sign (+).

## Data Availability

Data are contained within the article.
